# Multi-task deep latent spaces for cancer survival and drug sensitivity prediction

**DOI:** 10.1093/bioinformatics/btae388

**Published:** 2024-09-04

**Authors:** Teemu J Rintala, Francesco Napolitano, Vittorio Fortino

**Affiliations:** Institute of Biomedicine, School of Medicine, University of Eastern Finland, Kuopio 70210, Finland; Department of Science and Technology, University of Sannio, Benevento 82100, Italy; Institute of Biomedicine, School of Medicine, University of Eastern Finland, Kuopio 70210, Finland

## Abstract

**Motivation:**

Cancer is a very heterogeneous disease that can be difficult to treat without addressing the specific mechanisms driving tumour progression in a given patient. High-throughput screening and sequencing data from cancer cell-lines has driven many developments in drug development, however, there are important aspects crucial to precision medicine that are often overlooked, namely the inherent differences between tumours in patients and the cell-lines used to model them *in vitro*. Recent developments in transfer learning methods for patient and cell-line data have shown progress in translating results from cell-lines to individual patients *in silico*. However, transfer learning can be forceful and there is a risk that clinically relevant patterns in the omics profiles of patients are lost in the process.

**Results:**

We present MODAE, a novel deep learning algorithm to integrate omics profiles from cell-lines and patients for the purposes of exploring precision medicine opportunities. MODAE implements patient survival prediction as an additional task in a drug-sensitivity transfer learning schema and aims to balance autoencoding, domain adaptation, drug-sensitivity prediction, and survival prediction objectives in order to better preserve the heterogeneity between patients that is relevant to survival. While burdened with these additional tasks, MODAE performed on par with baseline survival models, but struggled in the drug-sensitivity prediction task. Nevertheless, these preliminary results were promising and show that MODAE provides a novel AI-based method for prioritizing drug treatments for high-risk patients.

**Availability and implementation:**

https://github.com/UEFBiomedicalInformaticsLab/MODAE.

## 1 Introduction

High-throughput sequencing of omics data can be used to characterize the molecular state of cells and tissues in both patient tumours and cancer cell-lines that are used to model them. Very large patient cohorts such as the cancer genome atlas (TCGA) have been used to study the molecular mechanisms underlying cancer progression and the remarkable heterogeneity of tumours, leading to the development of precision medicine approaches that target specific genomic aberrations to improve treatment outcomes (Chin *et al.* 2011). On the other hand, cancer cell-lines have been widely screened *in vitro* for sensitivity with large panels of anti-cancer compounds and thoroughly characterized in projects such as CCLE (Barretina *et al.* 2012, Ghandi *et al.* 2019), GDSC (Yang *et al.* 2013), and CTRP (Basu *et al.* 2013, Seashore-Ludlow *et al.* 2015). Large collections of screening results together with comprehensive molecular profiles of the cell-lines provide incredible resources for data-mining drug mechanisms and targeted therapies. However, the heterogeneity of cancer aberrations and the intrinsic differences between cancer cell lines and patient tumours has made it difficult to identify appropriate models for specific (sub-)types of cancer (Salvadores *et al.* 2020). In order to translate drug screening results to patient tumours it is very important to properly account for these differences (e.g. by matching each patient tumour to a cell-line model based on the similarity of their omics profiles). However, instead of directly establishing the best representative model for each cancer, the issue could be addressed as a domain adaptation problem to translate predictive models learned on cell-line omics profiles to the domain of patient tumour omics profiles. Recently, deep learning approaches have been shown to outperform traditional machine learning and domain adaptation techniques in this complex task (He *et al.* 2022). Accurately translated sensitivity screens could help prioritize drugs to a given group of omics profiles and reduce drug development costs for precision oncology.

To address the problem of transfer learning, Dincer *et al.* (2020) implemented an adversarial extension of the autoencoder model (AD-AE) to obtain a representation of data free from confounding factors between datasets. CODE-AE-ADV by He *et al.* (2022) used a more robust version of the adversarial autoencoder model by utilizing the Wasserstein distance as inspired by WGAN (Arjovsky *et al.* 2017, Gulrajani *et al.* 2017). CODE-AE-ADV also implemented shared and private embeddings for learning separate latent representations of the confounders in addition to the deconfounded representation shared between data domains. The goal of CODE-AE-ADV was to obtain a representation that contains only the shared characteristics of both cell-line and patient tumour omics profiles and to use this representation to learn a transferable model of drug sensitivity prediction. In addition to bulk omics, a similar transfer learning schema has also been applied to single-cell drug sensitivity by Chen *et al.* (2022). However, focusing solely on optimizing drug sensitivity may skew the shared embedding space towards features more informative to cancer cell lines than to patient cases. For a comprehensive cancer precision medicine approach, it is essential that the genomic embedding space that connects cell-line and patient tumour omics profiles, remains relevant to cancer patients. This can be achieved by incorporating additional learning tasks focusing on clinical aspects, such as survival data or molecular subtypes. This ensures the genomic data space accurately reflects patient information as well as cancer cell line information, potentially facilitating the identification of clinically actionable cancer subtypes. By studying patients with similar genomic profiles and drug sensitivities, and linking these to specific survival outcomes, we can attain a true precision medicine approach. To integrate these disparate objectives within machine learning we can use multi-task learning (MTL) approaches. MTL can be used to learn multiple tasks simultaneously instead of building separate models for each task or learning in stages. The combination of autoencoders for feature extraction and predictive models as part of MTL has been previously utilized for drug-sensitivity prediction (Chen *et al.* 2022) as well as survival prediction (Vo *et al.* 2021) separately in the context of cancer omics. In this article, we implement a novel end-to-end deep learning architecture extending the CODE-AE-ADV to include patient survival prediction and a joint learning scheme to learn a latent space that is suitable for prioritizing drugs for groups of patients based on their omics profiles.

## 2 Materials and methods

### 2.1 MODAE

#### 2.1.1 Autoencoder

The autoencoder consists of an encoder module $E:R^{d}\to R^{p}$ and a decoder module $D:R^{p}\to R^{d}$. We denote the input omics with **x**, the bottle-neck layer as z=E(x) and the reconstructed omics as $\hat{x}=D(z)$. The purpose of the autoencoder is to find a low dimensional representation $z\in R^{p}$ of the data in an unsupervised manner by minimizing the reconstruction loss


(1) $$L_{\text{rec}}(X)=\frac{1}{nd}\sum_{i=1}^{n}{\left|\left|{\hat{x}}^{\left(i\right)}-x^{\left(i\right)}\right|\right|}^{2}.$$

The input dimension *d* is included in the denominator in order to normalize the scales of different objectives for the MTL step (Equation (8)).

#### 2.1.2 Adversarial deconfounding autoencoder

To learn separate representations for confounders, we split the vector **z** into three parts: $z_{s}$ (shared), $z_{p}$ (patient tissue), and $z_{c}$ (cell-line). In contrast to He *et al.* (2022), we use a single encoder to learn all these representations by setting parts of **z** to **0** by applying the following function *f* before the decoder (i.e. $\hat{x}=D(f(z)$)):


(2) $$f_{j}(z_{j},s)=\{\begin{array}{cc} z_{j} & \text{if}\;j\in U\\ z_{j} & \text{if}\;j\in V\;\wedge \;s=0\\ z_{j} & \text{if}\;j\in W\;\wedge \;s=1\\ 0 & \text{otherwise}\; \end{array},$$

where *U* is the set of indices used for the shared representation, *V* is the set of indices for patients, and *W* is the same for cell-lines, and where s∈{0,1} is an indicator for whether a given sample is from a cell-line (and not from patient tissue). Supervised tasks use the shared part and we denote q=|U| as its dimension. An adversary aims to distinguish cell-lines from patients and the WGAN inspired autoencoder is forced to learn representations that minimize differences between the two data domains, which should lead to more reliable transfer of predictions. The adversary and the autoencoder are trained in alternating steps. In the first step the critic network $C:R^{q}\to R$ is trained to minimize the adversarial loss


(3) $$L_{\text{adv}}(Z_{s},s)=\frac{1}{n}\sum_{i=1}^{n}C(z_{s}^{\left(i\right)})(1-2s_{i}).$$

Then, the autoencoder is trained to minimize the sum of $L_{\text{rec}}$ and the deconfounding loss


(4) $$L_{\text{dc}}(Z_{s},s)=\frac{1}{\sum_{i=1}^{n}s_{i}}\sum_{i=1}^{n}C(z_{s}^{\left(i\right)})s_{i}$$

which is equivalent to the cell-line specific term of $L_{\text{adv}}$ with the sign reversed. $L_{\text{adv}}$ is an approximation of the Wasserstein distance between the distributions of the two sets of points in the latent representation (Arjovsky *et al.* 2017). In simpler terms, the critic or dataset detector network *C* is trained to estimate the patient-likeness of a given sample while the deconfounding autoencoder tries to minimize the distance of the cell-line sample distribution from the patient sample distribution. Furthermore, in order to satisfy the continuity assumptions of the WGAN model, we use a gradient penalty on $\nabla C(z_{s})$ similarly to He *et al.* (2022). However, our formulation of $L_{\text{adv}}$ and $L_{\text{bc}}$ differs from the equivalent $L_{\text{critic}}$ and $L_{\text{gen}}$ in that we use the shared representation $z_{s}$ while He *et al.* (2022) use a concatenation of shared and private representations and in our case the deconfounding loss is calculated on cell-lines instead of patient tissues.

#### 2.1.3 Deep Cox regression for survival modelling

A deep learning extension of the non-parametric Cox regression model can be obtained with the hazard function:


(5) $$\lambda (x)\propto e^{g(x)}=e^{A\left(f_{s}(E(x)),k\right)},$$

where $A:R^{q}\to R$ is a survival risk prediction network, $f_{s}:R^{p}\to R^{q}$ is a function that extracts the shared representation from **z**, and **k** is an optional vector of additional input covariates, such as patient age or sex. We used Efron’s method (Efron 1977) to account for tied survival times which can be quite common for large datasets or aggressive cancers. Hence, the loss function for training *A* is based on the adjusted Cox partial log-likelihood


(6) $$\begin{array}{c} L_{\text{surv}}=\frac{1}{\sum_{h\in H}|h|}\sum_{i=1}^{\left|H\right|}\left[\sum_{j\in h_{i}}g(x^{\left(j\right)})-r(X,h_{i},T)\right],\\ r(X,h_{i},T)=\sum_{l=0}^{|h_{i}|-1}\ln\left(\sum_{j:T_{j}\geq t_{i}}e^{g(x^{\left(j\right)})}-\frac{l}{\left|h_{i}\right|}\sum_{j\in h_{i}}e^{g(x^{\left(j\right)})}\right), \end{array}$$

where $h_{i}\in H$ is the set of indices of all events for which $T_{j}=t_{i}\forall j\in h_{i}$, *H* contains the sets of events for each unique time point *t_i_*, and $x^{\left(i\right)}$ is the vector of omics data for patient *i*. To improve the numerical stability of the calculations we utilized the log-sum-exp trick. The likelihood was divided by the number of observed events to maintain consistent scaling of the losses.

#### 2.1.4 Deep regression for drug sensitivity modelling

Drug sensitivity prediction was addressed as a multiple output regression problem. Since every drug-cell-line pair was not screened in CTRP, losses were computed on non-missing values only. In other words, we minimize the loss


(7) $$L_{\text{drug}}=\frac{1}{\left|O(R)\right|}\sum_{i,j\in O(R)}||{\hat{R}}_{ij}-R_{ij}|{|}^{2},$$

where **R** is a matrix of observed drug data that may contain missing values, *O* is a function that returns the set of indices of observed cell-line drug pairs, and $\hat{R}$ contains drug-sensitivity vectors **r**^(*i*)^ = $B(z_{s}^{\left(i\right)})$ computed for each cell-line *i* with the drug-sensitivity network $B:R^{q}\to R^{m}$, where *m* is the number of drugs.

#### 2.1.5 Joint MTL

To simultaneously learn to represent the molecular state, to find a shared deconfounded latent space, and to predict both patient survival and cell-line drug sensitivity from omics data we define the total loss as a weighted sum of the losses used for individual tasks as follow:


(8) $$L_{\text{MTL}}=\alpha_{1}L_{\text{rec}}+\alpha_{2}L_{\text{dc}}+\alpha_{3}L_{\text{surv}}+\alpha_{4}L_{\text{drug}}.$$

The weights $\alpha_{1-4}$ can be used to adjust the task priority and were tuned as hyper-parameters to explore different trade-offs as described in Section 3.2.

Algorithm 1. Multi-task training of MODAE

**Input:**  $X_{p}\;\text{patient tissue omics},$

$X_{c}\;$cell-line omics,

t patient survival time,

e patient survival event indicator,

K patient clinical data (e.g. age),

R cell-line drug-sensitivity matrix

**Require:**  $N_{\text{epochs}}$; network modules: A, B, C, D, E

1: **for**  $\text{epoch}=1\;\text{to}\;N_{\text{epochs}}$  **do**

2:  **for**  $X_{\text{mini}-p},X_{\text{mini}-c},t_{b},e_{b},K_{b},R_{b}\in$

 $\text{mini}-\text{batches}(X_{p},X_{c},t,e,K,R)$  **do**

3:    $Z_{p}=E(X_{\text{mini}-p})$

4:    $Z_{c}=E(X_{\text{mini}-c})$

5:    $Z=\text{concatenate}\_\text{samples}(Z_{p},Z_{c})$

6:    $s\;\text{is an indicator vector for}\;z^{\left(i\right)}\in Z_{c}$

7:    $Z_{\text{augmented}}=\text{concatenate}\_\text{features}(Z_{p},K_{b})$

8:    $\text{Update}\;A,B,D,E\;\text{with}\;L_{\text{MTL}}$

9:   **end for**

10:   **for**  adversarial epoch=1 to Nadv_per_AE  **do**

11:    **run** Critic training epoch

12:   **end for**

13: **end for**

To ensure robust training for the multi-task model, we implemented a pre-training procedure for each network module that corresponds to a specific task (Supplementary Algorithm 1). The autoencoder, critic network, deconfounding autoencoder, patient-survival network, and drug-sensitivity network can be trained in order before running the joint learning that updates the full model at each step (Supplementary Algorithm 2). In practice, skipping the first two steps and starting from the deconfounding autoencoder training worked better than pre-training each step. Pre-training of the deconfounding autoencoder was continued for 300 epochs, followed by 100 epochs each of patient survival and cell-line drug-sensitivity module training, after which the joint multi-task training was run for 300 epochs. Algorithm 1 shows the joint learning procedure which samples equally sized mini-batches from both datasets and updates the weights of all MODAE modules based on Equation (8). Since the number of patients and cell-lines can be different, we employed an oversampling strategy to generate the same number of mini-batches from both sample types for each epoch. Supplementary Fig. S1 shows the constituent losses for each epoch across training phases and cross-validation folds for a model selected by parameter search. We used the scaled exponential linear units (Klambauer *et al.* 2017) to increase the robustness of the joint model in place of more conventional batch-normalization. Furthermore, L2-norm regularization was applied on all the model weights, and dropout was applied after all layers except outputs and bottle-neck. The critic network *C* was trained in epochs that alternate with the autoencoder epoch in pre-training and multi-task epoch in joint training. Additionally, for each epoch of the deconfounding and joint training the adversary was trained for 5 epochs and used the Adam optimizer with recommended momentum parameters $\beta_{1}=0,\beta_{2}=0.9$ as suggested by Arjovsky *et al.* (2017) and Gulrajani *et al.* (2017).

### 2.2 Data and pre-processing

To perform a case-study on real patient data, we used cancer tissue data from the Swedish SCANB breast cancer cohort (Staaf *et al.* 2022) as a discovery dataset and the TCGA BRCA cohort as a validation dataset. To simplify the analysis, we focused on RNA-Seq data since the transcriptome has been shown to be the most relevant omic for drug sensitivity prediction (Costello *et al.* 2014). The SCANB data was already pre-processed to fragments per kilobase per million (FPKM) while pre-processed TCGA data downloaded with the *curatedTCGAData* R-package (Ramos *et al.* 2020) was in upper-quartile (UQ) normalized transcripts per million (TPM). To harmonize them, SCANB data was normalized to UQ TPM using the *edgeR* R-package and then both were log-transformed to ${\;\text{log}\;}_{2}(\text{TPM}+1)$. Cell-line transcriptome profiles were acquired from the DepMap portal (Corsello *et al.* 2020) which were already equivalently processed. Genes with average log-expression less than 1.0 in either the patient and cell-line dataset were excluded and the values in each dataset were independently centred and scaled to mean 0 and standard deviation 1. This yielded gene-expression datasets of 12 021 genes, 9206 patients for discovery, 1093 patients for validation, and 1293 cell-lines for discovery.

Survival data was obtained from Liu *et al.* (2018) who re-analysed TCGA clinical data to provide more accurate survival information. For drug sensitivity, we used CTRP which contains one of the most comprehensive drug-sensitivity datasets spanning 545 different drugs screened across 841 cell-lines of which 755 could be matched with transcriptomic profiles in CCLE (Basu *et al.* 2013, Seashore-Ludlow *et al.* 2015). CTRP data was downloaded using the *PharmacoGx* R-package (Smirnov *et al.* 2016). We decided to use the area above the curve (AAC) as the target drug-sensitivity value, since it is bounded in the range [0,1] and is defined even when some other common alternatives, such as IC50, are not. Performance was evaluated with 5-fold cross-validation stratified with respect to the survival event and time in the case of patient data. Since the Cox model depends on the order of the events (Equation (6)), event times were split into 10 equally sized quantiles after which the samples were evenly distributed into the folds based on the time quantile and event status. To account for the differences in time elapsed since TCGA and SCANB studies were conducted, the maximum follow-up time was set to 3000 days.

## 3 Results

### 3.1 Overview of MODAE

**Figure 1.**
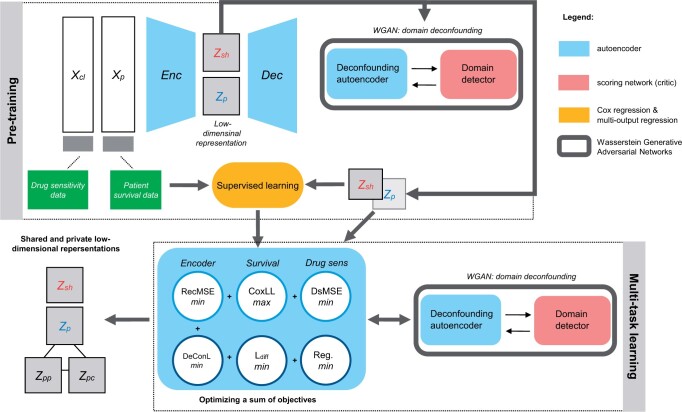
MODAE model diagram showing the essential structure and training steps. Cell-line omics profiles *X*_cl_ and patient omics profiles *X*_p_ are first encoded into two parts, the shared representation *z*_sh_ and private representation *z*_p_ which are further divided into private cell-line and private patient representations *z*_pc_ and *z*_pp_, respectively. The WGAN-based adversarial training adjusts the autoencoder with the help of a domain detector. Deconfounded shared representations are used for supervised training using drug-sensitivity and survival data. In a final training stage, all tasks are trained simultaneously by optimizing a sum of objectives.

We developed MODAE to integrate and deconfound patient tissue and cell-line samples for survival and drug sensitivity related feature extraction in a hybrid unsupervised-supervised fashion (see Fig. 1). Since survival and drug sensitivity are specific to their corresponding data domain, task specific models must be adapted to new target domains. MODAE achieves this by learning a shared representation of the molecular data which the task specific models can influence to mold it to be more useful for survival and drug sensitivity prediction. Differently from CODE-AE, we treat drug sensitivity prediction as a regression problem and perform MTL with supervised tasks together with the reconstruction error.

### 3.2 Trade-offs between evaluation metrics in random hyper-parameter search

**Figure 2.**
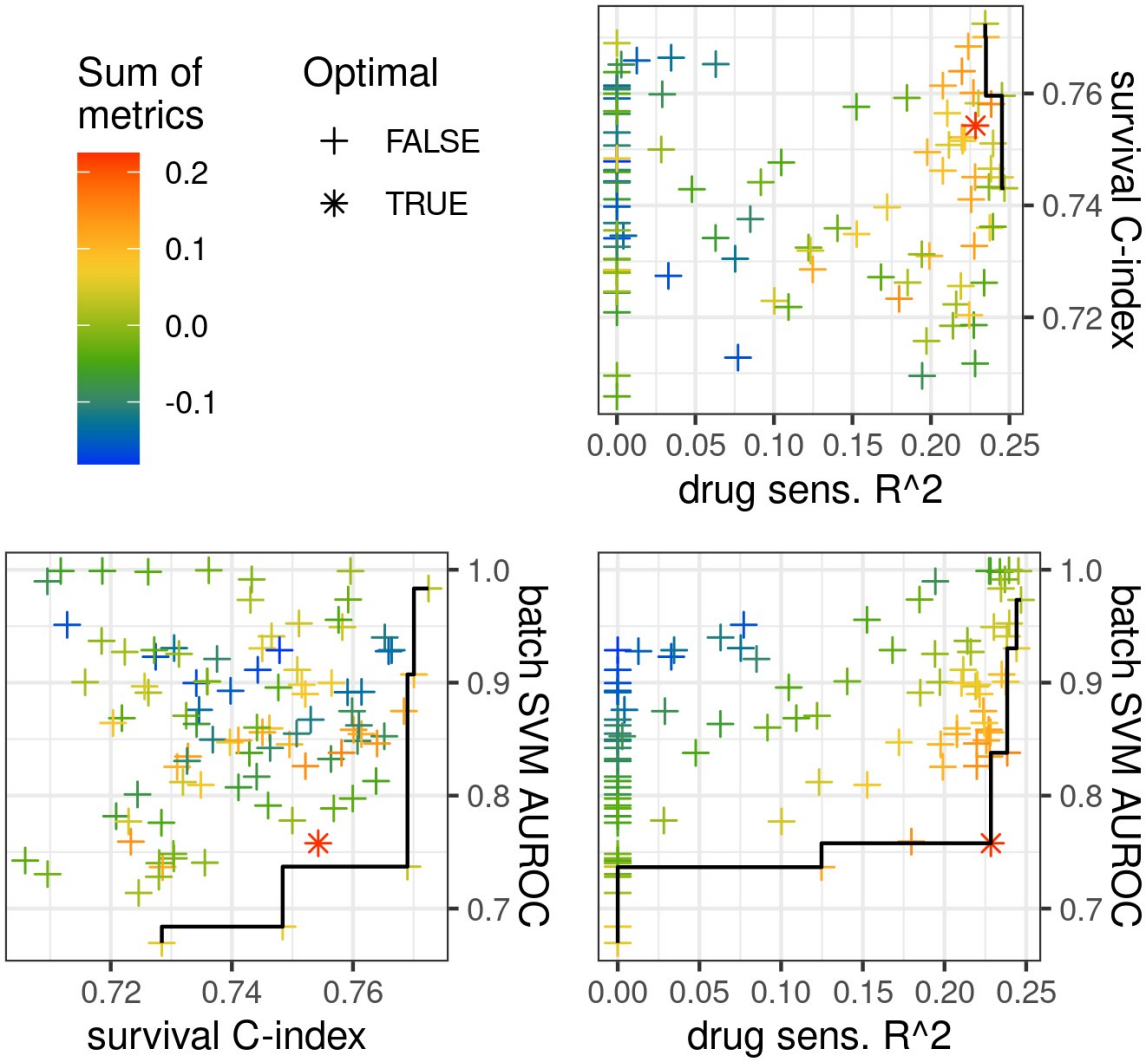
Pairwise scatter plots of key evaluation metrics. Each point represents one random parameter search result and the gradient represents the average sum of the test set survival concordance-index, top 10% drug sensitivity *R*^2^ quantile, and batch-effect defined as negative AUROC of a data domain detecting linear SVM. Drug-sensitivity performance in the plot was truncated at 0 since a portion of settings resulted in negative *R*^2^ suggesting a failure to learn useful models. The black line goes through points that are on the first Pareto front of each pairwise comparison. The optimal setting used for downstream analysis is highlighted by using an asterisk in place of a cross.

To search for the optimal hyper-parameters of MODAE, 100 iterations of random parameter search were performed. For the internal evaluation using 5-fold cross-validation in SCANB, the parameter search was carried out in a nested fashion by using one of the training folds as a validation set for parameter selection. Then for the test on external data (TCGA), the hyper-parameters were selected based on the 5-fold cross-validation results on SCANB. In either case, the selection of the best hyper-parameters is a multi-objective optimization problem. MODAE aims to optimize four different objectives: omics profile reconstruction, deconfounding, survival prediction, and drug sensitivity prediction. The random parameter-search was implemented to explore different trade-offs between these objectives by adjusting the learning rate, regularization, and number of hidden units associated with each module of MODAE as well as the weights assigned to the different objectives in Equation (8). Figure 2 shows the 5-fold average performance of each hyper-parameter setting: deconfounding performance in terms of AUROC achieved by a linear SVM in distinguishing the shared embeddings corresponding to different datasets, survival concordance in Harrell’s C-index (Harrell 1982), and 90th percentile sensitivity prediction in *R*^2^. We evaluated each drug prediction separately and focus only on top 10% of drug sensitivity predictions because we wanted to focus on drugs for which the models could explain a large portion of variance across the cell-lines. Settings that were better in at least one metric compared to the others form a Pareto front which indicates results with the best trade-offs. The model corresponding to the highest sum of these metrics was compared to baseline models in cross-validation and external datasets.

### 3.3 Baseline models

As baseline, we used elastic net regression with simple feature selection and PCA. For drug-sensitivity prediction, each baseline drug model was trained independently and implemented with the *scikit-learn* Python module (Pedregosa *et al.* 2011). For survival we used the penalized Cox model implementation provided in the *scikit-survival* Python module (Pölsterl 2020). The elastic net penalty and L1-ratio hyper-parameters were optimized via grid-search in nested cross-validation with the ranges $\left[10^{-3},10^{-1}\right]$ and [0.1,0.99], respectively. Feature selection was based on univariate regression models and the top-*k* approach, where the number of top predictors *k* was tuned as a hyper-parameter in the range [100,1000]. These models were trained independently of each other and are therefore not considered multi-task learners, but their purpose is to provide context for the performance of MODAE.

### 3.4 MODAE embeddings and performance comparison

**Figure 3.**
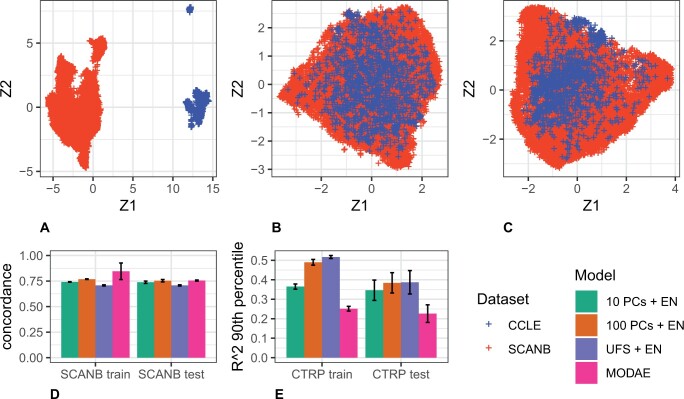
(**A**) UMAP visualization of the concatenated standardized gene-expression matrices corresponding to SCANB and CCLE. (**B**) UMAP visualization of the shared embeddings of SCANB and CCLE samples after pre-training the deconfounding autoencoder. (**C**) UMAP visualization of the shared embeddings of SCANB and CCLE samples after joint multi-task learning. (**D**) Mean survival concordance index of the proposed model and baselines cross-validated in SCANB. (**E**) Mean of the 90th percentile *R*^2^ of 545 drug sensitivity predictions cross validated in CTRP. Error bars represent standard deviation. PC: principal component; EN: elastic net regression; UFS: univariate feature selection; *R*^2^: coefficient of determination.

Figure 3A–C show UMAP (McInnes *et al.* 2018) visualizations of the differences between cell-lines and patients in the original gene-expression, the deconfounded shared representation learned in pre-training, and the final shared representation learned while compromising between multiple tasks. The first plot shows a clear difference between the datasets with no sample from either dataset overlapping the other. Meanwhile the second plot demonstrates the power of the deconfounding autoencoder where the samples from CCLE evenly overlap the samples from SCANB. The third plot shows that the compromise with MTL does not unravel the deconfounding effect learned during pre-training. Here all results are evaluated with the nested parameter search (i.e. the selected hyper-parameters can be different between folds). Figure 3D shows the survival concordance index of the models cross-validated in TCGA. All the models achieved concordance index around 0.75 with PCA based models performing better than univariate feature selection (UFS) and MODAE matching the best model in test set performance while showing slightly higher performance in the training set, but not to a large degree, which suggests that the model is adequately regularized to avoid overfitting. This result shows that MODAE achieves respectable performance even when burdened with deconfounding the predictive representation of differences between patient and cell-line profiles. Figure 3E shows the drug sensitivity prediction performance. Out of the three elastic net models, UFS performed the best, while 100 PCs was better than 10 PCs. MODAE performed significantly worse than the baselines, which suggests that the deconfounding process is hindering drug-sensitivity prediction. The 100 PC and UFS baseline models showed a significant degree of overfitting that was not present in MODAE. While the results shown here represent the average across best settings in each fold, it is consistent with Fig. 2 which shows that no individual setting of MODAE performed significantly better than the search average.

### 3.5 Prediction performance on external survival dataset

**Figure 4.**
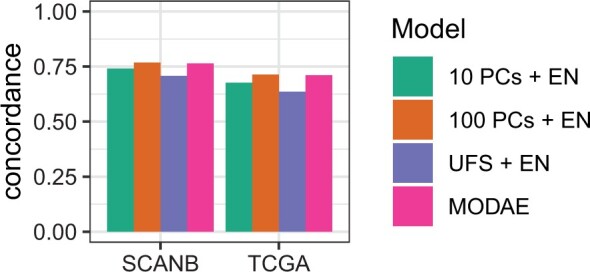
Survival prediction concordance index of MODAE and baseline models trained with SCANB and tested with TCGA BRCA.

**Figure 5.**
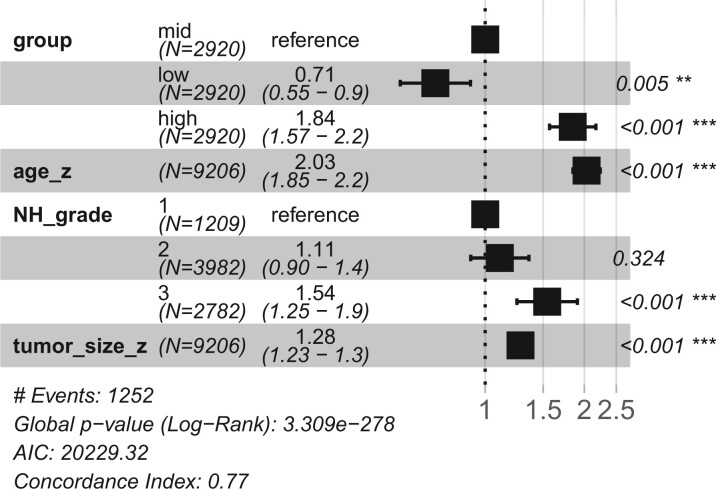
Hazard ratios of a CoxPH model with additional SCANB clinical variables and risk groups based on MODAE predictions.

**Figure 6.**
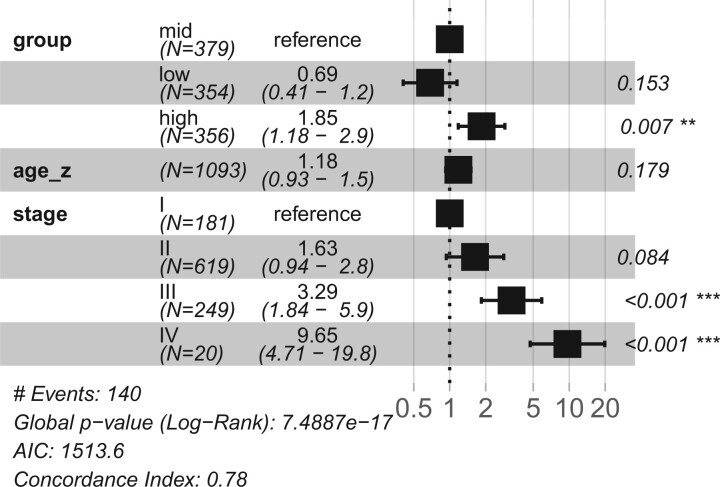
Hazard ratios of a CoxPH model with additional TCGA BRCA clinical variables and risk groups based on MODAE predictions.

We used the TCGA BRCA dataset as an external test set for survival predictions to see if the model predictions could be generalized to other datasets. The TCGA BRCA is a much smaller dataset but it has similar overall patient survival rate as the SCANB cohort with 16.3% in TCGA and 16.7% in SCANB. Since TCGA has much longer maximum follow up time, all observations beyond 3000 days were set to censored in both datasets to equalize both studies. Figure 4 shows the Harrell concordance index of MODAE as well as baseline models in the training set (SCANB) and the test set (TCGA). The external validation results reflect the cross-validation results with 100 PC EN and MODAE performing the best. The MODAE concordance index on the external dataset (0.710) was only slightly lower than on the internal test set (0.755) suggesting some losses in generalization. We also checked the proportional hazard assumption of MODAE by using the *survminer*-package in R. Supplementary Fig. S2 shows the summary of tests on the last hidden layer of the survival risk module of MODAE, which indicated that the values observed at the hidden layers were not dependent on time and hence the proportional hazard assumption held for the final model. Due to differences in clinical data not all relevant covariates could be considered while testing the model. Additionally, the concordance index is based on the order of risk scores and does not account for shifts in predictions. Therefore, to further assess the predicted risk scores, we stratified the patients according to the predicted risk scores given by MODAE by calculating three even quantiles (high, mid, low) of the SCANB risk scores and stratifying TCGA patients using the same quantiles. Figure 5 shows the hazard ratios of a CoxPH model fitted with the additional clinical variables and the risk category in SCANB and Fig. 6 shows the same for TCGA. In SCANB the group indicators, age *z*-score, and tumour size *z*-score were all significant while of the Nottingham histological grading only the third grade was significantly more predictive than the first grade. In TCGA only the high risk group from MODAE and the advanced tumour stages III and IV were significant compared to mid-risk and stage-I, respectively. Notably the coefficients associated with the low-risk and high-risk groups were similar between the datasets, but the larger size of the SCANB cohort resulted in more significant *P*-values.

### 3.6 Assessing transfer learning of drug sensitivity predictions

The expression of the target genes modulated by a given drug is expected to be dysregulated. Similarly to He *et al.* (2022), to assess the agreement of the predictions with this expectation, we divided the patients into resistant and sensitive groups based on the sensitivity predicted by MODAE and compared the target-gene expression between them. The resistant and sensitive groups were defined as sensitivity *z*-score below −1 or above 1, respectively. 96% of gene-targets were dysregulated in breast cancer patient data with significance level 0.05, which suggests agreement with sensitivity predictions. However, only 22% of them had a fold-change over 1.5, which may suggest some false positives, since the sheer number of patients in SCANB can lend significance even to small differences. Supplementary File S1 contains the log-fold changes between groups and *t*-test *P*-values for every drug and their targets listed in DrugBank.

## 4 Discussion

In this article we introduced a novel deep learning model to learn latent representations of omics data for predicting cancer patient survival and transfer of drug sensitivity predictions from cell-line screens to patients while adjusting for differences between the molecular profiles of these two sample types. We applied our model to a case study of breast cancer patients to illustrate its usefulness for precision medicine and patient stratification. The model performed well with survival prediction in cross-validation as well as external validation, suggesting that we succeeded in recapitulating clinically relevant patient information from their genomic profiles. However, the drug sensitivity predictions on cell-lines were less accurate compared to simple linear models. A major limitation of this breast cancer case-study is the domain adaptation with pan-cancer cell-lines in the CCLE dataset. We wanted to focus on breast cancer, since the SCANB dataset is an unparalleled resource for studying a single cancer type and TCGA BRCA is the single largest cancer dataset within TCGA. Unfortunately, there were not enough breast cancer cell-lines profiles available in CCLE to train reliable drug-sensitivity prediction models. In theory, the deconfounding autoencoder can learn to represent the other cancer types by using the private embeddings even if the other dataset only contains one type, however, the drug-sensitivity predictions are based on the shared embedding where cell-lines of different cancer types are all forced to a representation similar to breast cancer tissues. While the results are somewhat preliminary, they demonstrate that the model has the potential to aid in the discovery of new precision medicine therapies. In the future we aim to improve the drug-sensitivity model and extend the case-study to pan-cancer TCGA. Another interesting direction would be the use of drug-features (e.g. based on their chemical structures) since this could lead to targeted drug design for specific patient groups. Nevertheless, the proposed model could already be used to prioritize repositioning of drugs for high-risk patients.

## Supplementary Material

btae388_Supplementary_Data

## Data Availability

https://github.com/UEFBiomedicalInformaticsLab/MODAE.

## References

[btae388-B1] Arjovsky M , Chintala S, Bottou L. Wasserstein generative adversarial networks. Proc Int Conf Mach Learn 2017;70:214–23.

[btae388-B2] Barretina J , Caponigro G, Stransky N et al The cancer cell line Encyclopedia enables predictive modelling of anticancer drug sensitivity. Nature 2012;483:603–7. 22460905 10.1038/nature11003 PMC3320027

[btae388-B3] Basu A , Bodycombe NE, Cheah JH et al An interactive resource to identify cancer genetic and lineage dependencies targeted by small molecules. Cell 2013;154:1151–61. 23993102 10.1016/j.cell.2013.08.003 PMC3954635

[btae388-B4] Chen J , Wang X, Ma A et al Deep transfer learning of cancer drug responses by integrating bulk and single-cell RNA-seq data. Nat Commun 2022;13:6494. 36310235 10.1038/s41467-022-34277-7 PMC9618578

[btae388-B5] Chin L , Andersen JN, Futreal PA. Cancer genomics: from discovery science to personalized medicine. Nat Med 2011;17:297–303. 21383744 10.1038/nm.2323

[btae388-B6] Corsello SM, Nagari RT, Spangler RD et al Discovering the anti-cancer potential of non-oncology drugs by systematic viability profiling. Nat Cancer 2020;1:235–48. 32613204 10.1038/s43018-019-0018-6 PMC7328899

[btae388-B7] Costello JC , Heiser LM, Georgii E et al A community effort to assess and improve drug sensitivity prediction algorithms. Nat Biotechnol 2014;32:1202–12. 24880487 10.1038/nbt.2877 PMC4547623

[btae388-B8] Dincer AB , Janizek JD, Lee S-I. Adversarial deconfounding autoencoder for learning robust gene expression embeddings. Bioinformatics 2020;36(Suppl_2):i573–i582. 33381842 10.1093/bioinformatics/btaa796 PMC7773484

[btae388-B9] Efron B. The efficiency of Cox’s likelihood function for censored data. J Am Stat Assoc 1977;72:557–65.

[btae388-B10] Ghandi M, Huang FW, Jané-Vabuena J et al Next-generation characterization of the cancer cell line encyclopedia. Nature 2019;569:503–8. ISSN 0028–0836. 31068700 10.1038/s41586-019-1186-3 PMC6697103

[btae388-B11] Gulrajani I , Ahmed F, Arjovsky M et al Improved training of Wasserstein GANs. *Adv Neural Inf Process Syst* 2017;30:5767–77.

[btae388-B12] Harrell FE. Evaluating the yield of medical tests. JAMA 1982;247:2543. 7069920

[btae388-B13] He D , Liu Q, Wu Y, Xie L. A context-aware deconfounding autoencoder for robust prediction of personalized clinical drug response from cell-line compound screening. Nat Mach Intell 2022;4:879–92. 38895093 10.1038/s42256-022-00541-0 PMC11185412

[btae388-B14] Klambauer G , Unterthiner T, Mayr A, Hochreiter S. Self-normalizing neural networks. Adv Neural Inf Process Syst 2017;30:971–80.

[btae388-B15] Liu J , Lichtenberg T, Hoadley KA et al An integrated TCGA pan-cancer clinical data resource to drive high-quality survival outcome analytics. Cell 2018;173:400–16.e11. 10.1016/j.cell.201802.052 29625055 PMC6066282

[btae388-B16] McInnes L , Healy J, Melville J. UMAP: uniform manifold approximation and projection for dimension reduction. arXiv. 2018. https://arxiv.org/abs/1802.03426

[btae388-B17] Pedregosa F, Varoquaux G , Gramfort A et al Scikit-learn: machine learning in python. J Mach Learn Res 2011;12:2825–30.

[btae388-B18] Pölsterl S. Scikit-survival: a library for time-to-event analysis built on top of Scikit-learn. J Mach Learn Res 2020;21:1–6.34305477

[btae388-B19] Ramos M , Geistlinger L, Oh S et al Multiomic integration of public oncology databases in bioconductor. JCO Clinical Cancer Inf 2020;4:958–71. 10.1200/CCI.19.00119 PMC7608653 33119407

[btae388-B20] Salvadores M , Fuster-Tormo F, Supek F. Matching cell lines with cancer type and subtype of origin via mutational, epigenomic, and transcriptomic patterns. Sci Adv 2020;6:eaba1862. 10.1126/sciadv.aba1862 PMC7458440 32937430

[btae388-B21] Seashore-Ludlow B , Rees M, Cheah M et al Harnessing connectivity in a large-scale small-molecule sensitivity dataset. Cancer Disc 2015;5:1210–23. 10.1158/2159-8290.CD-15-0235 PMC4631646 26482930

[btae388-B22] Smirnov P , Safikhani Z, El-Hachem N et al PharmacoGx: an R package for analysis of large pharmacogenomic datasets. Bioinformatics 2016;32:1244–6. 26656004 10.1093/bioinformatics/btv723

[btae388-B23] Staaf J , Häkkinen J, Hegardt C et al RNA sequencing-based single sample predictors of molecular subtype and risk of recurrence for clinical assessment of early-stage breast cancer. NPJ Breast Cancer 2022;8:94. 35974007 10.1038/s41523-022-00465-3 PMC9381586

[btae388-B24] Vo T-H , Lee G-S, Yang H-J et al Survival prediction of lung cancer using small-size clinical data with a multiple task variational autoencoder. Electronics 2021;10:1396.

[btae388-B25] Yang W , Soares J, Greninger P et al Genomics of drug sensitivity in cancer (GDSC): a resource for therapeutic biomarker discovery in cancer cells. Nucleic Acids Res 2013;41(Database issue):D955–61. 23180760 10.1093/nar/gks1111 PMC3531057

