# Supplementary material for: Multi-task deep latent spaces for cancer survival and drug sensitivity prediction
Source: Bioinformatics. 2024 Sep 4;40(Suppl 2):ii182–9. doi: 10.1093/bioinformatics/btae388 (PMC11520233; doi:10.1093/bioinformatics/btae388)
Supplement: btae388_Supplementary_Data [file btae388_supplementary_data.zip › rintala_2024_supplementary.pdf]

Supplementary algorithms, figures, and tables for: Multi-task deep latent spaces for cancer survival and treatment response prediction

Teemu J. Rintala [1], Francesco Napolitano [2,*], and Vittorio Fortino [1,*]

[1] Institute of Biomedicine, University of Eastern Finland, 70210 Kuopio, Finland
[2] Department of Science and Technology, University of Sannio, 82100 Benevento, Italy
[*] To whom correspondence should be addressed:
vittorio.fortino@uef.fi,
francesco.napolitano@unisannio.it

1 Supplementary Algorithms

Algorithm 1 Pre-training epochs

Input: $\mathbf{X}_p$ patient tissue omics,
$\mathbf{X}_c$ cell-line omics,
$\mathbf{t}$ patient survival time,
$\mathbf{e}$ patient survival event indicator,
$\mathbf{K}$ patient clinical data (e.g., age),
$\mathbf{R}$ cell-line drug-sensitivity matrix
Require: $N_{\text{adv\_per\_AE}}$; network modules: A, B, C, D, E

procedure AUTOENCODER PRE-TRAINING EPOCH
    for $\mathbf{X}_{\text{mini-}p}, \mathbf{X}_{\text{mini-}c} \in \text{minibatches}(\mathbf{X}_p, \mathbf{X}_c)$ **do**
        $\mathbf{X}_{\text{mini}} = \text{concatenate\_samples}(\mathbf{X}_{\text{mini-}p}, \mathbf{X}_{\text{mini-}c})$
        Update E and D with L_{rec}
    end for
end procedure
procedure CRITIC TRAINING EPOCH
    for $\mathbf{X}_{\text{mini-}p}, \mathbf{X}_{\text{mini-}c} \in \text{minibatches}(\mathbf{X}_p, \mathbf{X}_c)$ **do**
        $\mathbf{X}_{\text{mini}} = \text{concatenate\_samples}(\mathbf{X}_{\text{mini-}p}, \mathbf{X}_{\text{mini-}c})$
        $\mathbf{s}$ is an indicator vector for $\mathbf{x}^{(i)} \in \mathbf{X}_{\text{mini-}c}$
        $\mathbf{Z} = E(\mathbf{X}_{\text{mini}})$
        Update C with $L_{\text{adv}}(\mathbf{Z}, \mathbf{s})$
    end for
end procedure
procedure BATCH CORRECTION PRE-TRAINING EPOCH
    for $\mathbf{X}_{\text{mini-}p}, \mathbf{X}_{\text{mini-}c} \in \text{minibatches}(\mathbf{X}_p, \mathbf{X}_c)$ **do**
        $\mathbf{X}_{\text{mini}} = \text{concatenate\_samples}(\mathbf{X}_{\text{mini-}p}, \mathbf{X}_{\text{mini-}c})$
        $\mathbf{s}$ is an indicator vector for $\mathbf{x}^{(i)} \in \mathbf{X}_{\text{mini-}c}$
        $\mathbf{Z} = E(\mathbf{X}_{\text{mini}})$
        Update C with $L_{\text{bc}}(\mathbf{Z}, \mathbf{s})$
    end for
    for adversarial epoch = 1 to $N_{\text{adv\_per\_AE}}$ **do**
        run CRITIC TRAINING EPOCH
    end for
end procedure
procedure SURVIVAL PRE-TRAINING EPOCH
    for $\mathbf{X}_{\text{mini}}, \mathbf{t}_b, \mathbf{e}_b, \mathbf{K}_b \in \text{minibatches}(\mathbf{X}_p, \mathbf{t}, \mathbf{e}, \mathbf{K})$ **do**
        $\mathbf{Z} = E(\mathbf{X}_{\text{mini}})$
        $\mathbf{Z}_{\text{augmented}} = \mathbf{Z} \oplus \mathbf{K}_b$
        Update A with L_{surv}
    end for
end procedure
procedure DRUG SENSITIVITY PRE-TRAINING EPOCH
    for $\mathbf{X}_{\text{mini}}, \mathbf{R}_b \in \text{minibatches}(\mathbf{X}_c, \mathbf{R})$ **do**
        $\mathbf{Z} = E(\mathbf{X}_{\text{mini}})$
        Update B with L_{drug}
    end for
end procedure

Algorithm 2 Pre-training network modules

Require: $N_{\text{AE-pte}}, N_{\text{C-pte}}, N_{\text{BC-pte}}, N_{\text{S-pte}}, N_{\text{D-pte}}$
Require: network modules: A, B, C, D, E
for epoch $= 1$ to $N_{\text{AE-pte}}$ **do**
    run AUTOENCODER PRE-TRAINING EPOCH
end for
for epoch $= 1$ to $N_{\text{C-pte}}$ **do**
    run CRITIC TRAINING EPOCH
end for
for epoch $= 1$ to $N_{\text{BC-pte}}$ **do**
    run BATCH CORRECTION PRE-TRAINING EPOCH
end for
for epoch $= 1$ to $N_{\text{S-pte}}$ **do**
    run SURVIVAL PRE-TRAINING EPOCH
end for
for epoch $= 1$ to $N_{\text{D-pte}}$ **do**
    run DRUG SENSITIVITY PRE-TRAINING EPOCH
end for

2 Supplementary figures and tables

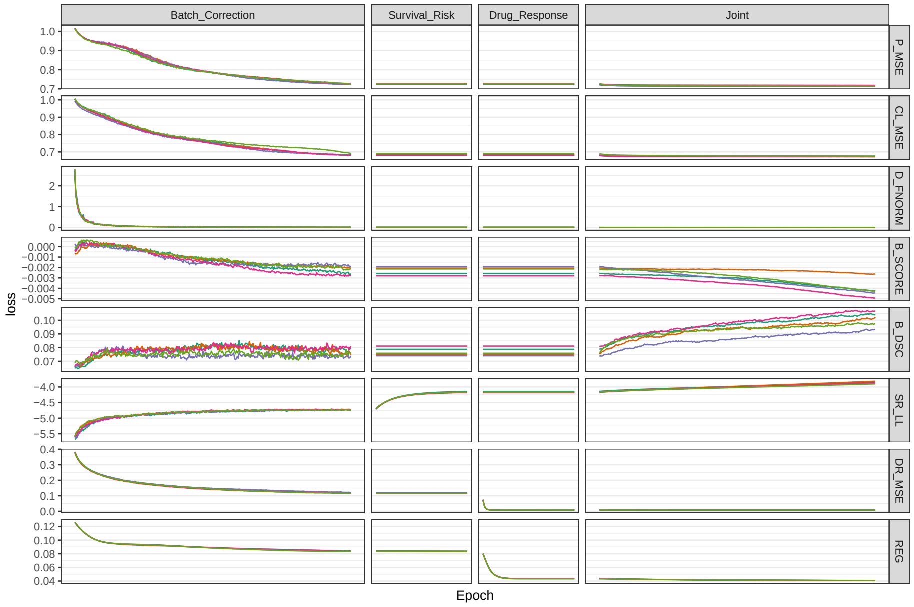

Figure 1: MODAE losses and additional metrics monitored over epochs. Different color lines represent different cross-validation folds. P_MSE = patient omic reconstruction mean squared error; CL_MSE = cell-line omic reconstruction mean squared error; D_FNORM = deconfounding norm penalty; B_SCORE = average critic score difference between patient and cell-line representations (corresponds to L_{adv}); B_DSC = dispersion separability criterion between patients and cell-line representations; SR_LL = Cox PH log-likelihood (higher is better); DR_MSE = drug-sensitivity regression mean squared error; REG = total losses from L2-norm penalty on model weights.

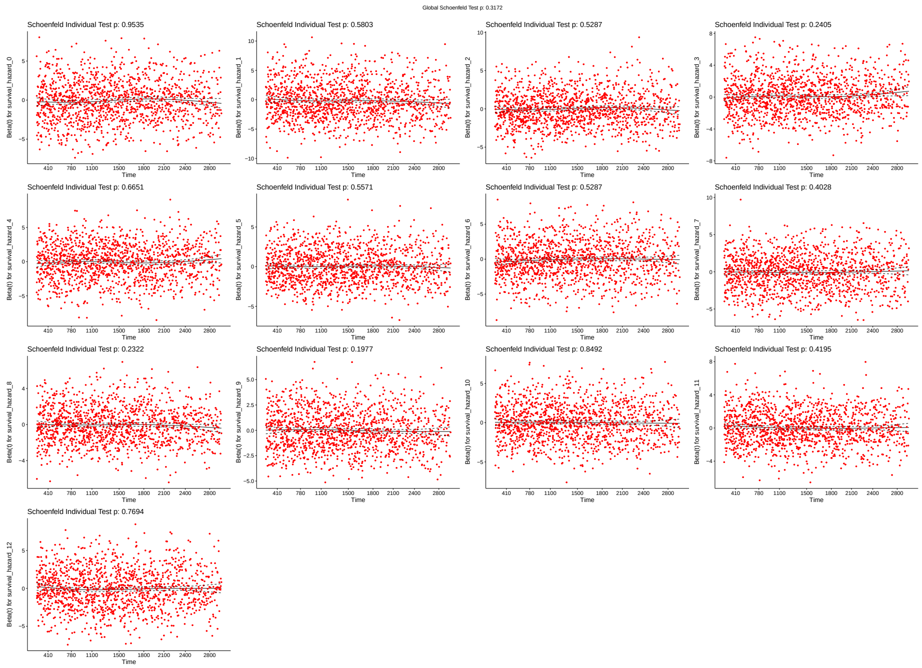

Figure 2: MODAE survival model Cox PH assumption tests based on last hidden layer values. No hidden unit was significantly associated with survival time, therefore the proportional hazards assumption holds.

Table 1: Gene-expression difference breast cancer patients stratified based on drug sensitivity. Genes identified of breast cancer drug targets. P-value was determined using the t-test.

drug	gene	log2FC	p_value
Abiraterone	CYP17A1	-0.015	0.001
Afatinib	EGFR	0.733	<0.001
Afatinib	ERBB2	-0.579	<0.001
Afatinib	ERBB4	-1.371	<0.001
Alisertib	AURKA	-1.154	<0.001
Alvocidib	EGFR	-0.704	<0.001
Alvocidib	PYGB	0.335	<0.001
Alvocidib	PYGM	0.072	0.001
Alvocidib	CDK7	-0.050	0.005
Alvocidib	CDK5	0.255	<0.001
Alvocidib	CDK9	0.286	<0.001
Alvocidib	CDK1	-1.084	<0.001
Alvocidib	CDK6	-0.665	<0.001

drug	gene	log2FC	p-value
Alvocidib	CDK4	-0.242	<0.001
Alvocidib	CDK8	-0.484	<0.001
Alvocidib	CDK2	-0.302	<0.001
Axitinib	FLT1	-0.115	<0.001
Axitinib	KDR	-0.058	0.019
Axitinib	FLT4	0.457	<0.001
Azacitidine	DNMT1	-0.343	<0.001
Bardoxolone methyl	NFKBIA	-0.116	<0.001
Belinostat	HDAC1	-0.174	<0.001
Bexarotene	RXRA	0.955	<0.001
Bexarotene	RXRB	0.175	<0.001
Bexarotene	RXRG	0.058	<0.001
Bortezomib	PSMB5	-0.127	<0.001
Bortezomib	PSMB1	-0.287	<0.001
Bosutinib	CDK2	-0.303	<0.001
Bosutinib	BCR	0.185	<0.001
Bosutinib	ABL1	0.348	<0.001
Bosutinib	LYN	-0.819	<0.001
Bosutinib	HCK	-0.357	<0.001
Bosutinib	SRC	0.133	<0.001
Bosutinib	MAP2K1	-0.143	<0.001
Bosutinib	MAP2K2	0.382	<0.001
Bosutinib	MAP3K2	-0.080	<0.001
Bosutinib	CAMK2G	-0.101	<0.001
Cabozantinib	KDR	-0.055	0.024
Cabozantinib	MET	-0.623	<0.001
Cabozantinib	RET	1.355	<0.001
Canertinib	EGFR	-0.698	<0.001
Cediranib	KDR	-0.056	0.023
Cerulenin	FASN	1.390	<0.001
Ciclopirox	ATP1A1	-0.037	0.05
Ciclosporin	CAMLG	0.281	<0.001
Ciclosporin	PPP3R2	-0.000	0.736
Ciclosporin	PPIA	-0.346	<0.001
Ciclosporin	PPIF	-0.189	<0.001
Cimetidine	HRH2	0.211	<0.001
Clofarabine	POLA1	-0.303	<0.001
Clofarabine	RRM1	-0.279	<0.001
Crizotinib	MET	-0.623	<0.001
Crizotinib	ALK	-0.140	<0.001
Crizotinib	ROS1	-0.131	<0.001
Crizotinib	MST1R	0.771	<0.001
Cyclophosphamide	NR1I2	-0.064	<0.001
Dabrafenib	BRAF	-0.085	<0.001
Dabrafenib	RAF1	-0.123	<0.001

drug	gene	log2FC	p-value
Dabrafenib	SIK1	0.395	<0.001
Dabrafenib	NEK11	0.600	<0.001
Dabrafenib	LIMK1	0.260	<0.001
Dacarbazine	POLA2	-0.410	<0.001
Dacarbazine	PGD	-0.092	<0.001
Daporinad	NAMPT	-0.337	<0.001
Dasatinib	BCR	-0.187	<0.001
Dasatinib	ABL1	-0.330	<0.001
Dasatinib	LYN	0.872	<0.001
Dasatinib	SRC	-0.136	<0.001
Dasatinib	EPHA2	-0.154	<0.001
Dasatinib	LCK	1.237	<0.001
Dasatinib	YES1	0.422	<0.001
Dasatinib	KIT	0.426	<0.001
Dasatinib	PDGFRB	-0.428	<0.001
Dasatinib	STAT5B	-0.122	<0.001
Dasatinib	ABL2	0.104	<0.001
Dasatinib	FYN	0.730	<0.001
Dasatinib	BTK	0.730	<0.001
Dasatinib	NR4A3	0.321	<0.001
Dasatinib	CSK	-0.086	0.005
Dasatinib	EPHA5	-0.003	0.585
Dasatinib	EPHB4	-0.281	<0.001
Dasatinib	FGR	0.390	<0.001
Dasatinib	FRK	-0.108	<0.001
Dasatinib	HSPA8	0.262	<0.001
Dasatinib	MAP3K20	-0.142	<0.001
Dasatinib	MAPK14	0.245	<0.001
Dasatinib	PPAT	0.345	<0.001
Decitabine	DNMT1	-0.342	<0.001
Decitabine	HDAC1	-0.174	<0.001
Decitabine	DNMT3A	-0.174	<0.001
Decitabine	DNMT3B	-0.789	<0.001
Dexamethasone	NR1I2	-0.064	<0.001
Dexamethasone	NR3C1	-0.117	<0.001
Dexamethasone	NR0B1	-0.033	0.056
Dexamethasone	ANXA1	-0.543	<0.001
Dexamethasone	NOS2	-0.018	0.21
Docetaxel	NR1I2	-0.065	<0.001
Docetaxel	TUBB1	0.018	0.067
Docetaxel	MAP2	-0.451	<0.001
Docetaxel	MAP4	0.156	<0.001
Docetaxel	MAPT	2.135	<0.001
Docetaxel	BCL2	0.840	<0.001
Doxorubicin	TOP2A	-1.180	<0.001

drug	gene	log2FC	p-value
Doxorubicin	NOLC1	-0.167	<0.001
Doxorubicin	TOP1	-0.275	<0.001
Doxorubicin	TOP2B	-0.162	<0.001
Elocalcitol	VDR	0.241	<0.001
Entinostat	HDAC1	-0.173	<0.001
Erismodegib	SMO	0.074	0.036
Erlotinib	EGFR	0.718	<0.001
Erlotinib	NR1I2	0.064	<0.001
Etoposide	TOP2A	-1.178	<0.001
Etoposide	TOP2B	-0.161	<0.001
Fingolimod	HDAC1	-0.174	<0.001
Fingolimod	S1PR5	-0.111	<0.001
Fingolimod	S1PR1	-0.043	0.183
Fingolimod	S1PR3	0.048	<0.001
Fingolimod	S1PR4	-0.469	<0.001
Fluorouracil	TYMS	-0.919	<0.001
Fluvastatin	HMGCR	-0.084	<0.001
Fluvastatin	HDAC2	-0.904	<0.001
Foretinib	KDR	-0.055	0.024
Foretinib	HGF	0.077	0.014
Fulvestrant	ESR1	2.923	<0.001
Gefitinib	EGFR	0.728	<0.001
Gemcitabine	RRM1	-0.279	<0.001
Gemcitabine	TYMS	-0.919	<0.001
Gemcitabine	CMPK1	-0.311	<0.001
Ibrutinib	BTK	-0.682	<0.001
Ifosfamide	NR1I2	0.064	<0.001
Imatinib	BCR	0.188	<0.001
Imatinib	ABL1	0.347	<0.001
Imatinib	KIT	-0.384	<0.001
Imatinib	PDGFRB	0.515	<0.001
Imatinib	NTRK1	0.155	<0.001
Imatinib	CSF1R	0.123	<0.001
Imatinib	PDGFRA	-0.322	<0.001
Imatinib	DDR1	0.588	<0.001
Imatinib	DDR2	-0.134	<0.001
Istradefylline	ADORA2A	0.163	<0.001
Istradefylline	ADORA1	0.263	<0.001
Itraconazole	CYP51A1	-0.031	0.165
Lapatinib	EGFR	-0.703	<0.001
Lapatinib	ERBB2	0.648	<0.001
Lenvatinib	FLT1	-0.112	<0.001
Lenvatinib	KDR	-0.056	0.023
Lenvatinib	FLT4	0.459	<0.001
Lenvatinib	RET	1.353	<0.001

drug	gene	log2FC	p-value
Lenvatinib	KIT	-0.383	<0.001
Lenvatinib	PDGFRA	-0.322	<0.001
Lenvatinib	FGFR1	0.229	<0.001
Lenvatinib	FGFR2	0.487	<0.001
Lenvatinib	FGFR3	0.799	<0.001
Lenvatinib	FGFR4	-0.202	<0.001
Linifanib	FLT1	-0.114	<0.001
Linifanib	KDR	-0.057	0.021
Linifanib	FLT4	0.457	<0.001
Linifanib	KIT	-0.377	<0.001
Linifanib	CSF1R	0.124	<0.001
Linifanib	FLT3	0.136	0.004
Linsitinib	INSR	0.326	<0.001
Linsitinib	IGF1R	1.342	<0.001
Lovastatin	HMGCR	-0.090	<0.001
Lovastatin	HDAC2	-0.912	<0.001
Lovastatin	ITGAL	-0.567	<0.001
Methotrexate	TYMS	-0.921	<0.001
Methotrexate	ATIC	-0.053	<0.001
Methotrexate	DHFR	-0.370	<0.001
Myricetin	JAK1	-0.072	<0.001
Myricetin	PIK3CG	-0.548	<0.001
Navitoclax	BCL2	0.840	<0.001
Navitoclax	BCL2L2	0.142	<0.001
Navitoclax	BCL2L2-PABPN1	0.243	<0.001
Navitoclax	BAD	0.868	<0.001
Nelarabine	POLA1	-0.303	<0.001
Neratinib	EGFR	-0.699	<0.001
Nilotinib	ABL1	0.347	<0.001
Nilotinib	KIT	-0.384	<0.001
Nintedanib	FLT1	-0.112	<0.001
Nintedanib	KDR	-0.056	0.023
Nintedanib	FLT4	0.459	<0.001
Nintedanib	LYN	-0.818	<0.001
Nintedanib	SRC	0.133	<0.001
Nintedanib	LCK	-1.197	<0.001
Nintedanib	PDGFRB	0.515	<0.001
Nintedanib	PDGFRA	-0.320	<0.001
Nintedanib	FGFR1	0.229	<0.001
Nintedanib	FGFR2	0.489	<0.001
Nintedanib	FGFR3	0.799	<0.001
Nintedanib	FLT3	0.139	0.003
Obatoclax	BCL2	0.840	<0.001
Olaparib	PARP1	-0.127	<0.001
Olaparib	PARP2	-0.380	<0.001

drug	gene	log2FC	p-value
Olaparib	PARP3	0.665	<0.001
Olaparib	AKR1C3	0.133	0.003
Omacetaxine mepesuccinate	RPL3	0.317	<0.001
Paclitaxel	NR1I2	-0.065	<0.001
Paclitaxel	TUBB1	0.018	0.067
Paclitaxel	MAP2	-0.451	<0.001
Paclitaxel	MAP4	0.155	<0.001
Paclitaxel	MAPT	2.135	<0.001
Paclitaxel	BCL2	0.841	<0.001
Pazopanib	FLT1	-0.115	<0.001
Pazopanib	KDR	-0.058	0.019
Pazopanib	FLT4	0.458	<0.001
Pazopanib	KIT	-0.379	<0.001
Pazopanib	PDGFRB	0.515	<0.001
Pazopanib	PDGFRA	-0.319	<0.001
Pazopanib	FGFR3	0.798	<0.001
Pazopanib	ITK	-1.238	<0.001
Pazopanib	FGF1	0.284	<0.001
Pazopanib	SH2B3	-0.227	<0.001
Procarbazine	MAOA	0.074	0.284
Prochlorperazine	DRD2	0.053	0.024
Prochlorperazine	HRH1	0.017	0.584
Prochlorperazine	ADRA1A	0.023	0.237
Prochlorperazine	ADRA2A	0.857	<0.001
Quizartinib	FLT3	0.137	0.004
Regorafenib	FLT1	-0.114	<0.001
Regorafenib	KDR	-0.057	0.02
Regorafenib	FLT4	0.458	<0.001
Regorafenib	ABL1	0.347	<0.001
Regorafenib	RET	1.349	<0.001
Regorafenib	BRAF	-0.086	<0.001
Regorafenib	RAF1	-0.123	<0.001
Regorafenib	EPHA2	0.184	<0.001
Regorafenib	KIT	-0.378	<0.001
Regorafenib	PDGFRB	0.516	<0.001
Regorafenib	FRK	0.130	<0.001
Regorafenib	NTRK1	0.155	<0.001
Regorafenib	PDGFRA	-0.318	<0.001
Regorafenib	DDR2	-0.132	<0.001
Regorafenib	FGFR1	0.230	<0.001
Regorafenib	FGFR2	0.490	<0.001
Regorafenib	TEK	0.113	<0.001
Regorafenib	MAPK11	0.377	<0.001
Ruxolitinib	JAK1	-0.072	<0.001
Ruxolitinib	JAK2	-0.382	<0.001

drug	gene	log2FC	p-value
Ruxolitinib	JAK3	-0.825	<0.001
Ruxolitinib	TYK2	0.225	<0.001
Selumetinib	MAP2K1	0.142	<0.001
Selumetinib	MAP2K2	-0.384	<0.001
Sildenafil	PDE5A	0.003	0.941
Sildenafil	PDE6G	-0.439	<0.001
Sildenafil	PDE6H	-0.012	0.011
Sildenafil	ODC1	-0.788	<0.001
Sildenafil	CD274	-0.952	<0.001
Simvastatin	HMGCR	0.088	<0.001
Simvastatin	HDAC2	0.883	<0.001
Simvastatin	ITGAL	0.571	<0.001
Sirolimus	MTOR	-0.040	<0.001
Sitagliptin	DPP4	0.322	<0.001
Sorafenib	FLT1	-0.115	<0.001
Sorafenib	KDR	-0.057	0.02
Sorafenib	FLT4	0.458	<0.001
Sorafenib	RET	1.351	<0.001
Sorafenib	BRAF	-0.086	<0.001
Sorafenib	RAF1	-0.123	<0.001
Sorafenib	KIT	-0.379	<0.001
Sorafenib	PDGFRB	0.516	<0.001
Sorafenib	FGFR1	0.229	<0.001
Sorafenib	FLT3	0.138	0.003
Sunitinib	FLT1	-0.113	<0.001
Sunitinib	KDR	-0.055	0.024
Sunitinib	FLT4	0.459	<0.001
Sunitinib	MET	-0.622	<0.001
Sunitinib	KIT	-0.377	<0.001
Sunitinib	PDGFRB	0.516	<0.001
Sunitinib	CSF1R	0.125	<0.001
Sunitinib	PDGFRA	-0.316	<0.001
Sunitinib	FLT3	0.138	0.003
Tacrolimus	FKBP1A	-0.457	<0.001
Tamoxifen	NR1I2	-0.064	<0.001
Tamoxifen	ESR1	2.927	<0.001
Tamoxifen	ESR2	-0.129	<0.001
Tamoxifen	PRKCA	-0.353	<0.001
Tamoxifen	SHBG	0.065	<0.001
Tamoxifen	EBP	-0.181	<0.001
Tamoxifen	AR	0.964	<0.001
Tamoxifen	KCNH2	0.127	0.001
Tamoxifen	ESRRG	0.278	<0.001
Tamoxifen	MAPK8	-0.148	<0.001
Tandutinib	FLT3	0.137	0.003

drug	gene	log2FC	p-value
Tandutinib	PDGFD	0.590	<0.001
Tanespimycin	HSP90AA1	-0.381	<0.001
Tanespimycin	HSP90AB1	-0.301	<0.001
Temsirolimus	MTOR	-0.040	<0.001
Teniposide	TOP2A	-1.177	<0.001
Thalidomide	CRBN	0.074	<0.001
Thalidomide	ORM1	0.452	<0.001
Tivantinib	MET	-0.625	<0.001
Tivozanib	FLT1	-0.111	<0.001
Tivozanib	KDR	-0.056	0.022
Tivozanib	FLT4	0.458	<0.001
Tivozanib	MET	-0.626	<0.001
Tivozanib	KIT	-0.384	<0.001
Tivozanib	PDGFRB	0.515	<0.001
Tivozanib	PDGFRA	-0.322	<0.001
Tivozanib	FGFR1	0.228	<0.001
Tivozanib	FLT3	0.137	0.004
Tivozanib	TEK	0.113	<0.001
Tivozanib	PTK6	0.911	<0.001
Topotecan	TOP1	-0.275	<0.001
Topotecan	TOP1MT	-0.058	0.017
Tosedostat	NPEPPS	0.194	<0.001
Tosedostat	LTA4H	-0.262	<0.001
Trametinib	MAP2K1	0.147	<0.001
Trametinib	MAP2K2	-0.388	<0.001
Tretinoin	RXRA	0.956	<0.001
Tretinoin	RXRB	0.175	<0.001
Tretinoin	RXRG	0.058	<0.001
Tretinoin	RARG	0.390	<0.001
Tretinoin	ALDH1A1	-0.324	<0.001
Tretinoin	GPRC5A	0.890	<0.001
Tretinoin	ALDH1A2	0.216	<0.001
Tretinoin	RARRES1	-1.769	<0.001
Tretinoin	RARA	1.091	<0.001
Tretinoin	RARB	-0.512	<0.001
Tretinoin	LCN1	0.011	0.034
Tretinoin	OBP2A	0.399	<0.001
Tretinoin	RBP4	0.337	<0.001
Tretinoin	PDK4	0.168	0.004
Tretinoin	CYP26A1	0.294	<0.001
Tretinoin	CYP26B1	-0.153	<0.001
Tretinoin	CYP26C1	0.013	<0.001
Tretinoin	HPGDS	0.395	<0.001
Trifluoperazine	DRD2	0.054	0.023
Trifluoperazine	ADRA1A	0.023	0.229

drug	gene	log2FC	p-value
Trifluoperazine	CALY	0.125	<0.001
Trifluoperazine	CALM1	-0.271	<0.001
Trifluoperazine	TNNC1	0.353	<0.001
Trifluoperazine	S100A4	-0.395	<0.001
Valdecoxib	PTGS2	-0.352	<0.001
Valdecoxib	CA2	-0.019	0.777
Valdecoxib	CA3	-0.105	0.018
Vandetanib	EGFR	0.725	<0.001
Vandetanib	RET	-1.358	<0.001
Vandetanib	TEK	-0.105	<0.001
Vandetanib	PTK6	-0.915	<0.001
Vandetanib	VEGFA	0.069	0.026
Veliparib	PARP1	-0.125	<0.001
Veliparib	PARP2	-0.379	<0.001
Vincristine	TUBB	-0.360	<0.001
Vincristine	TUBA4A	-0.378	<0.001
Vorapaxar	F2R	0.072	0.019
Vorinostat	HDAC1	-0.174	<0.001
Vorinostat	HDAC2	-0.903	<0.001
Vorinostat	HDAC3	-0.031	0.002
Vorinostat	HDAC6	0.164	<0.001
Vorinostat	HDAC8	0.018	0.142
